# Spectral pattern similarity analysis: Tutorial and application in developmental cognitive neuroscience

**DOI:** 10.1016/j.dcn.2022.101071

**Published:** 2022-01-15

**Authors:** Verena R. Sommer, Luzie Mount, Sarah Weigelt, Markus Werkle-Bergner, Myriam C. Sander

**Affiliations:** a Center for Lifespan Psychology, Max Planck Institute for Human Development, Berlin, Germany; b Department of Rehabilitation Sciences, TU Dortmund University, Germany

**Keywords:** Representational pattern similarity analysis, Electroencephalography (EEG), Time-frequency representations (TFR), Neural stability, Neural distinctiveness

## Abstract

The human brain encodes information in neural activation patterns. While standard approaches to analyzing neural data focus on brain (de-)activation (e.g., regarding the location, timing, or magnitude of neural responses), multivariate neural pattern similarity analyses target the informational content represented by neural activity. In adults, a number of representational properties have been identified that are linked to cognitive performance, in particular the stability, distinctiveness, and specificity of neural patterns. However, although growing cognitive abilities across childhood suggest advancements in representational quality, developmental studies still rarely utilize information-based pattern similarity approaches, especially in electroencephalography (EEG) research. Here, we provide a comprehensive methodological introduction and step-by-step tutorial for pattern similarity analysis of spectral (frequency-resolved) EEG data including a publicly available pipeline and sample dataset with data from children and adults. We discuss computation of single-subject pattern similarities and their statistical comparison at the within-person to the between-group level as well as the illustration and interpretation of the results. This tutorial targets both novice and more experienced EEG researchers and aims to facilitate the usage of spectral pattern similarity analyses, making these methodologies more readily accessible for (developmental) cognitive neuroscientists.

## 1 Introduction

A key notion in cognitive neuroscience is the concept of neural representation (Kriegeskorte and Kievit, 2013). It assumes that information is represented in neural activity. Incoming sensory information, for example, initiates a neural activation cascade, thus translating the percept into a neural code that corresponds to the perceived information (Tulving, 2007). Hence, neural representation is a concept that links the physical world to the way it is mentally experienced (cf. Bain, 1874; Churchland, 1986; Koch, 2004), indicated by a systematic relationship between features of the world and observed neural activity (Poldrack, 2020). The specificity with which experiences are encoded into neural representations is thought to be directly related to cognition, in particular how accurately and precisely aspects of the experience can later be remembered (Rissman and Wagner, 2012). Specifically, the *stability* of neural representations across time as well as their *distinctiveness* from other representations are considered to be crucial for memory success (e.g., Kobelt et al., 2021; Kuhl et al., 2012; Lu et al., 2015; Xue, 2018). Understanding how information is represented in the brain and how the quality of neural representations influences cognition are major goals in cognitive neuroscience (for related reviews, see Rissman and Wagner, 2012; Xue, 2018). Cognitive abilities improve across childhood and adolescence (e.g., Keresztes et al., 2018; Schneider, 2015; Shing et al., 2010; Weigelt et al., 2014), suggesting an enhancement of neural functioning that enables the formation of increasingly high-quality neural representations. However, developmental studies to date have only rarely looked at cognition through the lens of neural representations (cf. Cohen et al., 2019; Fandakova et al., 2019). In the following, we present current approaches on how to study neural representations and delineate their role in cognition, taking the example of episodic memory performance. We present arguments in favor of a representational perspective in developmental cognitive neuroscience and provide a step-by-step tutorial that will detail all necessary steps to conduct multivariate neural pattern similarity analyses on time–frequency-resolved EEG data.

In practice, neural representations are measured as patterns of neural activity, for example, during stimulus perception or imagination. It should be noted that there are ongoing debates on whether these neural activity patterns permit being interpreted as neural representations in the philosophical sense (cf. Baker et al., 2021). Nevertheless, content-specific neural activation patterns can be identified with various neuroimaging methods (cf. Ishai et al., 2000; Kuhl et al., 2012; Michelmann et al., 2018; Xue, 2018), which neuroscientists use to investigate neural representations and the factors influencing whether and how well information is successfully encoded into neural activity. Patterns of neural activity can be compared across conditions using representational similarity analysis (RSA; Edelman, 1998; Kriegeskorte et al., 2008; Kriegeskorte and Kievit, 2013). RSA quantifies the distance between neural representations in multidimensional space by, for example, determining correlations of the underlying activity patterns (Kriegeskorte et al., 2008; see Fig. 2). For instance, by correlating the neural activity patterns evoked by repetitions of a given stimulus, one can assess the stability, i.e., the self-similarity, of the stimulus representation over time (e.g., Kobelt et al., 2021; Xue et al., 2010). Correlations between the activity patterns in response to different stimuli can be taken as a measure of the similarity (or its inverse, distinctiveness) across neural representations (e.g., Davis et al., 2014; Sommer et al., 2019). For example, representations of similar content, such as different face stimuli, are more similar to each other, i.e., show a higher correlation, than those of relatively distinct content, such as faces and houses (cf. Kriegeskorte et al., 2008). Overall, RSA is a versatile tool to study neural representational properties that shape cognition.

Multivariate pattern analysis approaches such as RSA are more commonly adopted in the analysis of functional magnetic resonance imaging (fMRI) data. But pattern analysis approaches have gained additional traction in recent years for time-resolved brain recordings like magneto- and electroencephalography (M/EEG) as well (Carlson et al., 2019, Fahrenfort et al., 2018, Jafarpour et al., 2013). In addition to representing information in the two dimensions space and activation magnitude, neural activity is continuous, recurrent, and highly dynamic. Thus, time is also an important aspect for neural information coding, allowing multiple neural networks to coexist in the same space (Cohen, 2011). Using multivariate methods in M/EEG data has revealed critical insights concerning, for example, object perception (e.g., Contini et al., 2017; Teng et al., 2017), decision-making (e.g., Bode et al., 2012), and memory (e.g., Kerrén et al., 2018; Lu et al., 2015). Furthermore, the high temporal resolution of such data enables examination of the similarity of neural patterns at different time points within stimulus presentation trials, allowing one to identify when, and for how long, specific information is represented in the brain (King and Dehaene, 2014). To sum up, tools for analyzing the similarity of multivariate neural activity patterns measured with M/EEG offer great potential for studying the properties and temporal dynamics of neural representations.

The excitability of neural populations fluctuates rhythmically, resulting in oscillatory electrophysiological activity (Buzsáki and Draguhn, 2004, Singer, 1999, Tiesinga et al., 2008, Wang, 2010). Such rhythmic neural activity is thought to enable communication and integration within and across brain networks (Fries, 2015, Varela et al., 2001). Oscillatory activity is considered to be crucial for virtually every domain of cognition including long-term memory, which we use as example domain for the current tutorial (cf. Düzel et al., 2010; Fell and Axmacher, 2011; Hanslmayr and Staudigl, 2014; Sander et al., 2020). Research over the past two decades or so has identified rhythmic neural activity in various frequency bands as being critical for successful memory operations: It has been suggested that the hippocampus rapidly binds information through synchronized theta (~ 7 Hz) and gamma (> 30 Hz) oscillations (Lisman and Jensen, 2013, Rutishauser et al., 2010, Staudigl and Hanslmayr, 2013), whereas neocortical systems form long-term representations mediated by desynchronization of alpha (~ 10 Hz) and beta (~ 15 Hz) rhythms (Griffiths et al., 2019, Hanslmayr et al., 2012, Staudigl et al., 2015). Overall, theoretical considerations and empirical evidence (Axmacher et al., 2010, Lisman and Jensen, 2013) as well as simulations (Akam and Kullmann, 2010, Parish et al., 2021) indicate that neural activity on various time scales, i.e., across different frequencies, is coordinated in order to represent diverse contents (e.g., Whittingstall and Logothetis, 2009).

Although RSA is increasingly applied to EEG data, the majority of studies to date use it to compare spatiotemporal activity patterns (e.g., from event-related potentials), that is, the activation amplitudes across stimulus presentation time and electrodes (e.g., Chan et al., 2011; Fellner et al., 2020; Lu et al., 2015; Schaefer et al., 2011). In view of the recognized significance of rhythmic neural activity across a wide range of frequencies for cognitive processes, we extend previous approaches to pattern similarity in EEG here by implementing a pipeline that computes the similarity of time–frequency representations (TFRs) in children and adults (Sommer et al., 2019; for other approaches of RSA on spectral (intracranial) EEG patterns, see Michelmann et al., 2018; Staresina et al., 2016).

Many cognitive abilities including episodic memory advance across childhood and adolescence (Graf and Ohta, 2002, Li et al., 2004, Ngo et al., 2018) as relevant brain regions and functions mature (Casey et al., 2000, Keresztes et al., 2017, Ofen, 2012, Ofen et al., 2007, Tang et al., 2018). The observation that performance in many tasks improves across childhood, particularly with regard to the formation and retention of memories, indicates an increasing quality of the underlying neural representations (Bauer, 2015). For example, the ability to bind different features of an event into a unique memory representation and the capacity to keep similar memories separate are crucial competences that develop during childhood (Lee et al., 2016, Ngo et al., 2018). While age-related differences in representational properties are a relatively longstanding topic in neurocognitive aging research – in particular the hypothesis and evidence regarding neural dedifferentiation (Carp et al., 2011, Kobelt et al., 2021, Koen and Rugg, 2019, Li et al., 2001, Li et al., 2000, Park et al., 2004, Park et al., 2010) – childhood development has only recently been investigated from a representational perspective, and mainly with fMRI so far (cf. Cohen et al., 2019; Fandakova et al., 2019; O’Hearn et al., 2020). Accordingly, RSA is not yet a commonly utilized approach in developmental cognitive neuroscience, especially not for M/EEG studies. In adults, evidence is accumulating that neural distinctiveness and neural stability facilitate cognitive performance and change in the course of aging (Kobelt et al., 2021, Koen et al., 2020, Koen et al., 2019, Lu et al., 2015, Sommer et al., 2019, Xue, 2018), suggesting that representational properties may also play an important role in understanding cognitive development during childhood. RSA offers an excellent tool to study neural representations, and furthermore, EEG is a widely applicable tool, even for infants, thus offering the great potential to explore new research avenues and advance the understanding of neurocognitive development. The tutorial we provide here along with the accompanying data and code makes multivariate EEG pattern similarity analyses readily accessible for a broad audience of (developmental) researchers, nurturing the wider adoption of such approaches in developmental cognitive neuroscience.

**Fig. 1:**
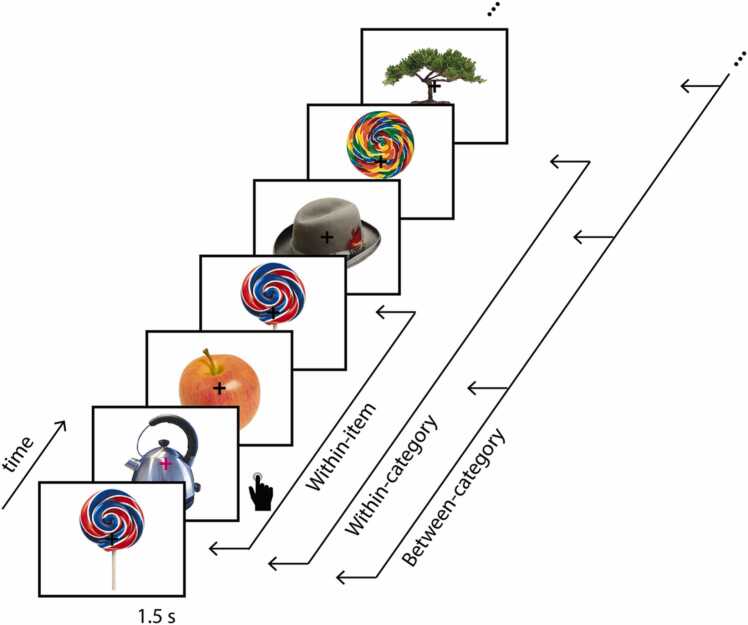
Overview of the encoding phase of the memory task paradigm (Sommer et al., 2021) and representational similarity levels. In the encoding task, objects were sequentially presented, and participants were asked to press a button whenever the fixation cross changed color. The sample dataset contains trials of two repetitions of two exemplars from each object category. Within-item similarity is the similarity of the neural patterns elicited by seeing identical objects. Within-category similarity is the similarity of the neural patterns evoked by different exemplars from the same object category. Between-category similarity is the mean pairwise similarity of the neural patterns evoked by all of the different object categories. Both within-category and between-category similarity are also called between-item similarity.

In the following, we provide a comprehensive tutorial guiding through the steps of time-resolved spectral pattern similarity analysis (cf. Sommer et al., 2019) to compute (and plot) the stability and distinctiveness of neural representations derived from TFRs, their statistical comparison using cluster-based permutation analysis implemented with FieldTrip (Maris and Oostenveld, 2007, Oostenveld et al., 2011), and the association with (memory) performance. The MATLAB code is publicly accessible and executable with the accompanying sample EEG dataset from ten children and ten young adults who participated in a memory study (Sommer et al., 2021; Fig. 1).

## 2 Representational similarity analysis on spectral EEG data

### 2.1 Tutorial overview

**Fig. 2:**
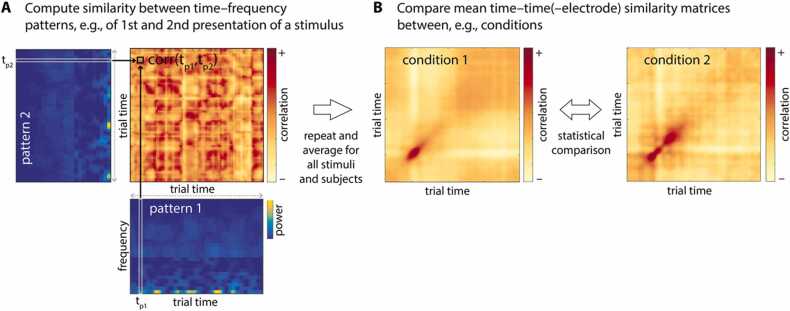
Illustration of spectral EEG pattern similarity analysis. A. Representational similarity is operationalized as pairwise correlations of the frequency patterns at each trial time point, separately for each electrode and subject. B. The resulting time–time similarity matrices contain the individual similarity at all trial time point combinations and can be averaged across trials, electrodes, and/or participants, and compared between conditions or groups, for instance. To assess differences in similarity patterns across conditions or groups, non-parametric cluster-based permutation statistics can be applied.

This tutorial provides comprehensive step-by-step instructions that detail all necessary computations to conduct multivariate neural pattern similarity analyses on time–frequency-resolved EEG data (as recently applied in Sommer et al., 2019, see Fig. 2 below for a schematic illustration). Furthermore, we demonstrate how cluster-based permutation statistics (Maris and Oostenveld, 2007) can be used to ascertain differences in neural patterns across representational levels and age groups.

Figure adapted from Sommer et al. (2019).

As exemplary data analysis pipelines, we demonstrate how to assess neural stability of representations (**within-item pattern similarity**) across stimulus repetitions and neural distinctiveness in response to different stimuli (**between-item pattern similarity**). Furthermore, we contrast within-item similarity and between-item similarity to examine whether the neural representations are item-specific (cf. Kobelt et al., 2021; Xue et al., 2010; Zheng et al., 2018).

The tutorial is accompanied by a sample dataset comprising EEG data from children and adults (Sommer et al., 2021) as well as custom-written MATLAB code interfacing with the open-source FieldTrip toolbox (Oostenveld et al., 2011). The tutorial has been tested on MATLAB R2016b, R2019b, R2020b and FieldTrip-20180709 and FieldTrip-20210507 (not provided but available at https://www.fieldtriptoolbox.org/download/) and does not require high-performance computing power. In addition to the input data (single-subject TFRs), we also provide all intermediate outputs such that all analysis steps can be executed independently of each other. The data and code are publicly available under https://osf.io/jbrsa/.

### 2.2 Sample dataset

The sample dataset includes EEG data from ten 7–9-year-old children (6 female, 4 male) and ten 18–30-year-old adults (5 female, 5 male) during the encoding phase of an episodic memory study (Fig. 1; for a detailed description of the task and data preprocessing, see Sommer et al., 2021). The data made available stem from a subsample of the original participants and selected conditions of the original experiment that allow the interested reader to run this tutorial. In particular, the task length differed between children and adults in the original study. Here, we included an identical number of trials for all age groups (except for subject-specific differences in the number of excluded trials during preprocessing). Furthermore, for both children and adults, we excluded trials that are irrelevant for the current tutorial pipeline (e.g., additional stimulus repetitions). Included trials stem from the encoding part of the paradigm in which participants viewed images of objects from 40 different categories (e.g., lollipops, hats). Categories are represented by two different exemplars and all exemplars are presented twice. Stimuli were presented successively at the center of a computer screen on a white background. A central fixation cross was superimposed on the objects and remained on the screen throughout the task. The stimulus presentation lasted 1500 ms with an inter-stimulus-interval jittered between 1500 and 2000 ms. The stimulus order was pseudorandomized such that 3–10 stimuli (from other categories) appeared between repetitions of the same item and at least 5 items were presented in-between different exemplars from the same category. Participants were instructed to attend to the objects but to fixate on the cross in order to minimize eye movements. To ensure that participants attended to each trial, they performed a target detection task in which they were asked to press a button whenever the fixation cross changed its color from black to magenta. Trials with targets were excluded from all analyses and are also not included here.

EEG data provided here are preprocessed, artifact-free single-subject single-trial TFRs that were obtained via a multitaper approach (for details, see below). Therefore, the number of trials included in the individual TFRs varies across participants based on the number of excluded trials due to artifacts in the EEG data. The TFRs are the basis for all representational similarity analyses described in this tutorial. Furthermore, for the sample dataset, all intermediate outputs for within-item and within-category similarity (but not between-category similarity) are provided as well. For a detailed description of the enclosed files in the dataset, see the Wiki on the Open Science Framework (OSF) repository (https://osf.io/jbrsa/wiki/).

### 2.3 Setup for running the RSA tutorial

To run the analyses on the sample dataset, download the code repository and data to your computer (https://osf.io/jbrsa/). In addition, download the FieldTrip toolbox (https://www.fieldtriptoolbox.org/download/). All steps of the analyses are implemented in separate (wrapper) functions. Each function receives a config struct as input that sets all adjustable specifications for the respective analysis. All implemented steps are listed in Table 1.

**Table 1:** All analysis steps included in the tutorial along with the respective functions to run them, the required settings for the config input, the required data, and the output that is returned and/or saved. You need the individual TFRs to start; all other required data can be produced by previous steps. For the sample dataset, all intermediate data are also provided so you can also start with any later step. For details on each function, see code documentation.

Analysis step + function	Required settings in config	Required data (input)	Saved data (output)
**Step 1: Run RSA for each subject** step1_rsa_get_sim_matrices	– subjects – type – pdat.tfr – pdat.rsa	individual time–frequency EEG data (TFRs)	individual similarity matrices
**Step 2: Compute similarity grand average** step2_rsa_group_ga	– subjects – group – type – pdat.rsa – pdat.ga	individual similarity matrices	grand average similarity matrices
**Step 3: Plot average similarity matrix** step3_plot_sim_matrices	– group – type^a^ – pdat.ga – pdat.fig	grand average similarity matrices	group average time–time similarity plot, diagonal
**Step 4: Test for item specificity (within-item versus between-item similarity)** step4a_sim_comparison_1st_level	– subjects – group – pdat.tfr – pdat.rsa – pdat.stat – pdat.ga	individual similarity matrices	first-level statistics
step4b_sim_comparison_2nd_level		– first-level statistics – electrode layout	second-level statistics
step4c_sim_comparison_3rd_level		*for both age groups:* – first-level statistics – second-level statistics – individual similarity matrices	– third-level statistics – individual mean similarities
**Step 5: Plot clusters** step5_plot_clusters	– group – pdat.ga – pdat.stat – pdat.fig – pdat.tfr	– grand average similarity matrices – first-level statistics – second-level statistics – electrode layout	plots showing cluster dimensions
**Step 6: Plot similarity comparison** step6_plot_sim_comparison	– pdat.ga – pdat.fig	*for both age groups:* – individual mean similarities	plots comparing similarities and age groups
**Step 7: Correlate with memory performance** step7_correlation_with_behavior	– pdat.ga – pdat.beh – pdat.fig	– individual mean similarities – individual mean item memory	– correlation results – scatter plot

a If this is not specified, both within-item and within-category similarity will be plotted.

In principle, the analysis steps need to be executed one after the other because the previous step’s output is usually required as the next step’s input. However, for the sample dataset, all intermediate outputs are provided as well, so that each step can be run individually (for within-item and within-category similarity). To run the functions in the intended order, we suggest using the script config_and_run_rsa.m. Here, you can adjust the config input that is required for all analysis steps and run them one by one, without adjusting the individual functions. Specifically, you need to configure your path settings under config.pdat (in code section "Path configurations"), specifying where to find the data, the FieldTrip toolbox, where to save the results etc. Furthermore, the RSA itself requires configuration (in code section "RSA configurations") namely which data should be analyzed (age group and individual subject IDs) and which representational level (type, see below). By default, the results are saved into the specified folder(s), but you may also run any of the steps without saving the output by setting config.save to false (0). No specific folder structure is required for the input and output data.

### 2.4 A note on the input: time–frequency representations of EEG data

In the sample dataset, the TFRs comprise a large range of frequencies, namely from 2 to 125 Hz. However, the input data are not restricted to a specific frequency range or resolution but can be varied according to research questions and hypotheses. There are several methods to achieve frequency decomposition that are not part of the current tutorial (for details, see Abbate et al., 2002; Cohen, 2003; Debnath, 2003). The enclosed single-subject TFRs of the sample dataset were derived using a multitaper approach. For low frequencies (2–20 Hz), Hanning tapers were used with a fixed width of 500 ms, resulting in frequency steps of 2 Hz. For higher frequencies (25–125 Hz), discrete prolate spheroidal sequences (DPSS) tapers were used with a width of 400 ms in steps of 5 Hz with seven Slepian tapers, resulting in ± 10 Hz smoothing. We used trial lengths of –0.6 to 2 s relative to stimulus onset. In this way, we obtained TFRs for each trial and electrode, resulting in 4-dimensional power spectra for each participant (trial × electrode × frequency × time).

The data are provided in accordance with the FieldTrip data structure as required for use of the tutorial pipeline (see https://www.fieldtriptoolbox.org/development/datastructure/).

### 2.5 Computing estimates of representational similarity

RSA can be used to investigate neural representations at different levels (see Fig. 1). The similarity of the neural activation patterns elicited by the *same* stimulus input (within-item similarity; also called self-similarity) is an indicator of the stability of the neural representation across repetitions. And the similarity or dissimilarity of activation patterns elicited by *different* stimulus inputs (between-item similarity) is an indicator of how distinctively these stimuli are represented. Such between-item representational similarity can be assessed between items from the same broader stimulus category (within-category similarity) and between items from different stimulus categories (between-category similarity).

To measure within-item representational similarity, the respective stimulus items have to be presented at least twice while brain activity is recorded. A common approach is to assess the neural pattern similarity between these first and second stimulus presentations (e.g., Kobelt et al., 2021). Another possibility is to present the stimuli more than twice and measure within-item similarity as the mean similarity across all repetitions (e.g., Lu et al., 2015). With regard to between-item similarity, one may be interested in the similarity of two or more stimuli, such as all stimuli that were presented during the experiment (also called global similarity) which may belong to one or different categories (Davis et al., 2014, Kobelt et al., 2021, Sommer et al., 2019).

Note that category membership is to some degree variable and often differs between studies. In the current dataset, exemplars from the same object category (e.g., different hats) are defined as belonging to one category, and different objects (e.g., hats, trees) are defined as different categories (Sommer et al., 2021). Other studies may select more specific (e.g., cowboy hats, oaks) or more superordinate categories (e.g., clothes, plants, or inanimate and animate objects).

Overall, there is a large methodological variety in how RSA can be used to assess representational properties such as neural stability and distinctiveness (e.g., Carp et al., 2011; Davis et al., 2014; Lu et al., 2015). Not all of these different approaches to within-item and between-item similarity are implemented in the current tutorial or feasible in the provided sample dataset (for details of what is implemented, see below). But the general logic and procedures resemble each other across different approaches, such that the pipeline can be adjusted easily and extended to allow the desired similarity analyses.

### 2.6 Implementation of within-item similarity (stability)

To compute RSA on the subject level, the function step1_rsa_get_sim_matrices is called. For within-item similarity, adjust the config.type input to specify the desired similarity level to 'within-item' (you may use the config_and_run_rsa script to configure and run each analysis step). For each subject, step1_rsa_get_sim_matrices invokes the function self_rsa that loads the TFRs, i.e., the spectral power across time and frequency at all electrodes and for all presented stimulus trials (trial × electrode × frequency × time). The function then selects the TFRs of the trials to be correlated, namely the spectral patterns evoked during the first and second presentations of items from all object categories (for simplicity, first exemplars only). This is done based on the trial information provided with the individual EEG data. Before computing the similarity, data are log-transformed and the background noise spectrum is removed from the TFRs to counter the effect of intrinsically high correlations between frequency patterns due to the 1/frequency characteristic of the EEG power spectrum (Schönauer et al., 2017). For this, an approach from the better oscillation detection (BOSC) framework is applied (Caplan et al., 2001; Kosciessa et al., 2020; Whitten et al., 2011; for discussion see also Allefeld et al., 2016; Cai et al., 2016) which is called via the function subtract_mean_noise_spectrum.

At the core of this pattern similarity analysis is the correlation of individual TFRs that is implemented in the function spectral_rsa called by self_rsa. Correlations are computed separately at each electrode between two time–frequency pattern matrices (pattern 1 and pattern 2; see Fig. 2A), here with 31 frequency bins from 2 to 125 Hz (see above), and 326 time points from 0.6 s before stimulus onset (0) to 2 s after stimulus onset. Specifically, the frequency vectors at each time point *t*_*p1*_ of the first pattern are correlated with the frequency vectors at each time point *t*_*p2*_ of the second pattern. Thus, we obtain a correlation coefficient for each *t*_*p1*_ × *t*_*p2*_ combination. If not specified otherwise, Pearson correlation is used. The resulting correlation matrix (time × time) is then Fisher-*z* transformed and returned. It represents the similarity of the two spectral patterns at all time point combinations. This procedure is repeated for all electrodes (for adults, these are 60 and for children 64 scalp electrodes) resulting in an electrode–time–time similarity matrix for each pair of correlated stimulus trials. Note that the number of trials varies between participants after preprocessing due to differences in EEG data quality and depends on your choice of type of RSA.

The resulting category × electrode × time × time similarity matrices are represented in a FieldTrip data structure for time–frequency data (see https://www.fieldtriptoolbox.org/development/datastructure/). Since having two time dimensions is not a valid data type, we denote one of the time dimensions as frequency and thus “fake” a frequency dimension such that FieldTrip treats the data like TFRs. In addition to the usual data and metadata fields required by FieldTrip, additional information about the original trials and object categories that were correlated in the RSA are also saved in the data structure. The function step1_rsa_get_sim_matrices then saves the individual similarity data in the specified output folder.

Before moving on to averaging the data, we highly recommend to carefully inspect (e.g., plot) some parts of the similarity results already on the trial level (not implemented in the current tutorial). This helps to get an impression of the overall level and variance of similarity in the analyzed data set and to potentially identify interesting patterns that may get lost with averaging similarity across trials.

In the next step, step2_rsa_group_ga computes and saves the grand averages of the individual similarity matrices. That is, the similarity matrices for each subject are averaged across items, resulting in a channel × time × time similarity matrix containing the respective representational similarities independent of the individual items or categories that were compared with each other. These average similarity matrices of each subject are combined in one data structure (subject × channel × time × time, with one time dimension again denoted as frequency; see above) and saved to the specified output folder.

**Fig. 3:**
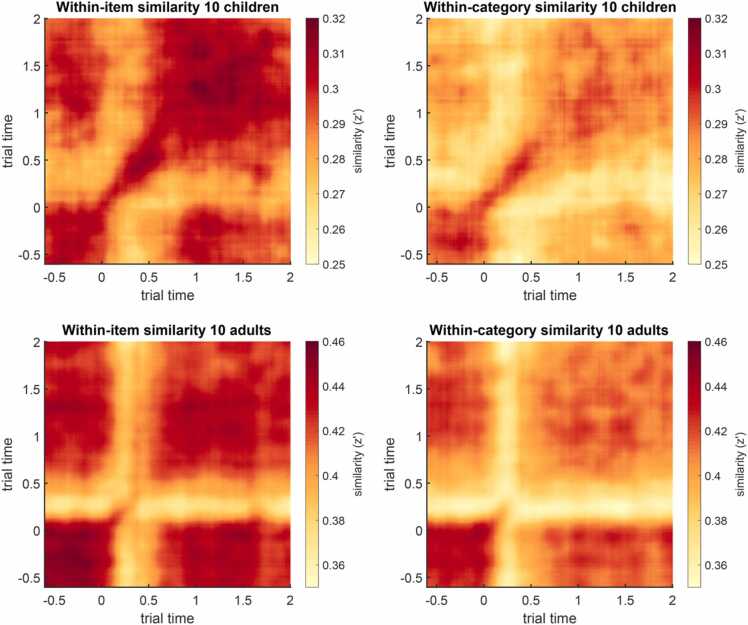
Time–time pattern similarity matrices of within-item similarity (left) and between-item (within-category) similarity (right), averaged across trials, electrodes, 10 children (CH; top), and 10 young adults (YA, bottom). Similarity is measured in Fisher-z transformed Pearson correlation coefficients (z’). These figures can be created with step3_plot_sim_matrices. Note that the color scales differ between age groups.

**Fig. 4:**
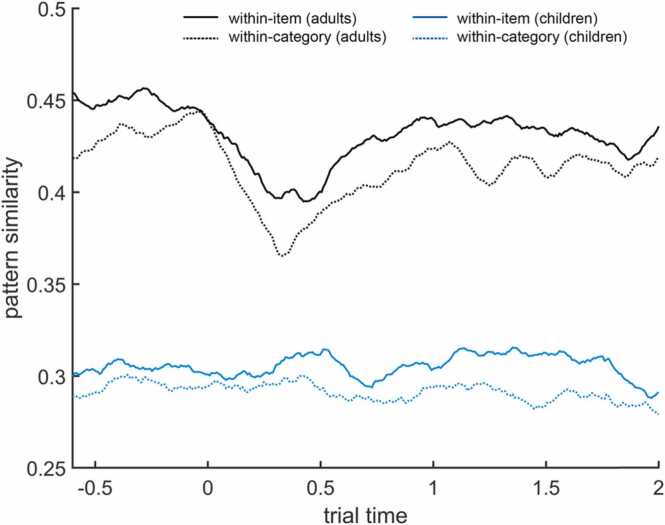
Diagonals of the time–time pattern similarity matrices (see Fig. 3) for within-item similarity (solid line) and within-category similarity (dotted line) in children (blue) and adults (black). These plots can be created with step3_plot_sim_matrices (separately for children and adults).

The group mean similarity matrices (averaged across all electrodes) and the diagonals of the mean similarity matrices can be plotted using step3_plot_sim_matrices (see Figs. 3 and 4). The diagonals show the similarity of the respective spectral patterns at identical time points. As elevated similarities often occur at and around the diagonal, one may want to plot the diagonals to better illustrate group or condition comparisons (see also Sommer et al., 2019). In the sample dataset, the pattern similarity values in adults are overall much higher than in children.

### 2.7 Implementation of between-item similarity (distinctiveness)

Between-item similarity can be computed for items from the same object category (within-category similarity) or for items from different object categories (between-category similarity), which can be selected by specifying config.type as 'within-cat' or 'between-cat', respectively, when running step1_rsa_get_sim_matrices. The trials to be correlated are selected with self_rsa or betw_cat_rsa, which are called by step1_rsa_get_sim_matrices for within-category or between-category similarity, respectively. Before computing the similarity, the data are log-transformed and the background noise spectrum is removed from the TFRs using BOSC (see above). Within-category similarity is computed as the correlation between spectral patterns evoked by the first and second exemplars of each object category (for simplicity, first presentations only). Between-category similarity is computed as the average similarity between each category and all other categories (for simplicity, first presentations of first exemplars only). This means that in within-category RSA (as in within-item RSA), exactly two TFRs for each object category are correlated with each other. Specifically, for *n* categories and, e.g., 60 electrodes, 60 × *n* time–time correlation matrices are computed for each subject. In contrast to that, between-category RSA computes all pairwise combinations of all available categories (except self-similarity), resulting in 60 × *n*-1 × *n*-1 time–time correlation matrices. Thus, between-category RSA requires considerably more computations and correspondingly takes longer to run. Again, spectral_rsa is called and runs the core computation of correlating the TFRs. In the next steps, the grand averages of the resulting similarity matrices are computed in step2_rsa_group_ga and plotted using step3_plot_sim_matrices (same as above).

The diagonal of a similarity matrix divides the matrix into an upper and a lower triangle (above and below the diagonal, respectively). These triangles are not necessarily mirrored. For within-category similarity, for instance, each time point of the neural pattern during presentation of the first exemplar (trial 1) is compared to each time point of the neural pattern of the second exemplar (trial 2). If the beginning of trial 1 shows high similarity to the end of trial 2, this high similarity would appear off-diagonal in one of the triangles and not in both. The mirrored point in the other triangle would instead show the similarity of the end of trial 1 and the beginning of trial 2. However, for between-item similarities in which all pairwise comparisons are computed (here, between-category similarity), each correlation of the frequency vectors at two time points is actually computed twice, thus appearing on both sides of the diagonal. For example, the similarity of the responses to stimulus X and stimulus Y is computed twice but in different orders (similarity(X,Y) and similarity(Y,X)), namely once when X is compared to all other stimuli, and once when Y is compared to all other stimuli. The individual similarity matrices are not identical, but when the mean similarity is computed across all stimulus combinations, the resulting mean similarity matrix is mirrored across the diagonal. In these cases, one of the triangles suffices for plotting and subsequent analyses (cf. Sommer et al., 2019).

### 2.8 Statistical comparison of RSA matrices

Depending on the research questions and the level of comparison, we use different statistical tests, which are mainly implemented in FieldTrip (Oostenveld et al., 2011). To test for differences in representational similarity matrices, the major tool are non-parametric cluster-based random permutation statistics that account for the multiple comparison problem (Maris and Oostenveld, 2007; see also Fields and Kuperberg, 2020). Univariate two-sided, dependent or independent *t*-statistics are calculated for the time–time similarity matrices at all electrodes. Clusters are formed by grouping neighboring channel × time × time samples with a *p*-value below 0.05 (spatially and temporally). The respective test statistic is then determined as the sum of all *t*-values within a cluster. We use the Monte Carlo method to compute the reference distribution for the summed cluster-level *t*-values. To derive the reference distribution under the null hypothesis that there is no difference between groups/conditions, samples are repeatedly assigned into arbitrary groups and the *t*-tests are computed between these random groups and summed within the respective clusters. Finally, the summed *t*-values for a given cluster derived from the true group comparison is compared against the reference distribution of summed *t*-values from the same cluster derived from the random assignments.

### 2.9 Implementation

The computations are implemented in FieldTrip using the function ft_freqstatistics for time–frequency data (for a detailed tutorial, see https://www.fieldtriptoolbox.org/tutorial/cluster_permutation_freq/). As these statistical tests are intended for frequency-resolved data, we again “fake” TFRs by denoting one of the time dimensions of the electrode–time–time similarity matrices as frequency. FieldTrip functions receive a configuration input cfg that specifies the desired computations. Depending on the applied statistics, the output stat contains the *t*-maps, *p*-values, cluster dimensions etc. for all channel × time × time coordinates.

#### 2.9.1 Comparison between representational levels (item and category specificity)

If the neural patterns we measure with a given brain recording technique truly represent a specific content that is presented at the time, we would expect representational similarity between related contents (e.g., from the same stimulus category) to be higher than between less related stimuli (e.g., from different categories). Accordingly, several studies – especially those concerned with age-related differences in neural differentiation (Koen and Rugg, 2019, Li et al., 2001) – have measured neural representational (category) specificity as within-category similarity corrected for (i.e., subtracting) between-category similarity (e.g., Carp et al., 2011; Kobelt et al., 2021; Koen et al., 2019). In analogy, the specificity of neural *item* representations has been defined as higher item stability than representational similarity to other, similar items and thus assessed as the difference between within-item and within-category similarity (Kobelt et al., 2021, Xue et al., 2010). As such, the assessment of item specificity is a measure that combines neural stability and neural distinctiveness by quantifying or testing their difference. In this tutorial, we implement item specificity by directly testing within-item similarity against within-category similarity for each participant (first-level analysis). (Alternatively, we could also compute item specificity matrices by subtracting within-category similarity from within-item similarity and testing them against zero.) Subsequently, we test the *t*-values from the first-level analysis against zero to examine whether the differences are reliable on the group level (second-level analysis). For group comparison (third-level analysis), see section 2.4.2. Equivalently, testing for category specificity would involve testing within-category similarity against between-category similarity (not implemented here).

Note that, in addition to the stimulus content (e.g., the presented item or category), other factors may influence their neural representational similarity, for instance their temporal distance within the experiment. Such confounding factors need to be identified and corrected for.

### 2.10 Implementation of first-level (within-subject) analysis

Contrasting individual within-item similarity and within-category similarity matrices for all specified participants is implemented in the function step4a_sim_comparison_1st_level. Here, the FieldTrip input struct cfg is configured to specify the statistical test run with ft_freqstatistics. For each item, the item’s pattern similarity to itself across repetitions is contrasted against the item’s pattern similarity to the other item from the same object category using two-sided paired sample *t*-tests. The resulting *t*-maps can be considered as point-wise effect size measures of the difference between the representational levels. The *t*-maps of all specified subjects are concatenated and the stat output is returned and saved as 1st_level_stat to the specified output folder.

### 2.11 Implementation of second-level (within-group) analysis

In step4b_sim_comparison_2nd_level, the *t*-maps of the first-level analysis are tested against zero using two-sided independent samples *t*-tests, controlling for multiple comparisons by conducting cluster-based random permutation (500 ×) tests. The output stat2 contains the positive and negative cluster statistics and the channel × time × time coordinates of the identified clusters and is saved as 2nd_level_cluster_stat (for discussions of how the results should (not) be interpreted, see Maris and Oostenveld, 2007; Maris, 2012; Sassenhagen and Draschkow, 2019; and https://www.fieldtriptoolbox.org/faq/how_not_to_interpret_results_from_a_cluster-based_permutation_test/). Clusters are positive or negative based on the direction of the effect, which in this case is determined by the order in which within-item and between-item similarity were contrasted during the first-level analysis. In the provided dataset and with the current settings, the analysis identifies 2861 positive and 271 negative clusters for the child sample and 1788 positive and 137 negative clusters for the adult sample. Of these clusters, 2 positive clusters for children and 1 positive cluster for adults exceed the 97.5th percentile (*p*s < 0.025) for their respective reference distribution, indicating significant effects in both age groups. Here, positive clusters indicate that within-item similarity is significantly higher than within-category similarity (suggesting item specificity). Note that the number of permutations determines how small the *p*-values can be. With 500 permutations, *p*-values cannot be smaller than 0.002.

**Fig. 5:**
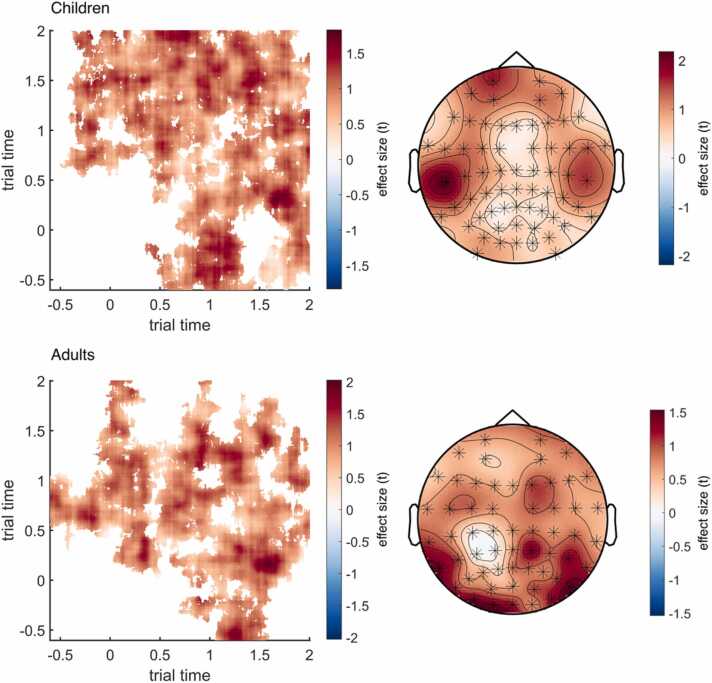
Visualization of effect sizes (*t*-values) in clusters identified to show item specificity (i.e., reliable differences between within-item and between-item similarity) in children (top) and adults (bottom). Left: Effect sizes within time × time cluster dimensions, averaged across significant electrodes. Right: Topographic representations of effect sizes across electrodes, averaged over significant time points. Highlighted channels (asterisks) are included in the cluster. Note that different EEG systems were used for children and adults, resulting in different electrode layouts. These images can be created with step5_plot_clusters.

**Fig. 6:**
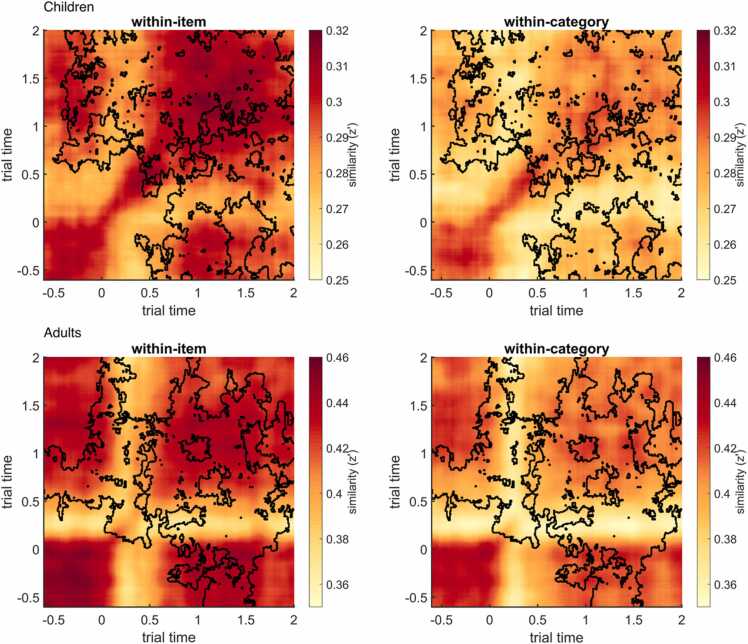
Pattern similarity matrices (identical to Fig. 2) plus outlines of identified clusters (see Fig. 5) showing at what time–time coordinates within-item (left) and within-category (right) similarities may show reliable differences, averaged across electrodes, for children (top) and adults (bottom). These images can be created with step5_plot_clusters.

The identified clusters span over a wide range of time–time combinations and over all electrodes (see Fig. 5), suggesting widespread differences in within-item and within-category similarity and thus highly item-specific neural representations. The identified cluster dimension can be used to extract similarity values from those channel × time × time coordinates in which differences were shown to be reliable (see Fig. 6).

#### 2.11.1 Comparison between age groups

Testing whether age groups differ in their neural representational properties can be approached in different ways. One straightforward approach is a cluster-based permutation analysis, similar to the one described above, directly contrasting the similarity data of participants of different ages. This could indicate cortical regions and time–time points at which age groups might show reliable pattern similarity differences. However, the interpretation of such age effects require careful consideration because they can potentially emerge due to many unspecific age differences, for instance, in skull thickness which substantially influences EEG signal (Frodl et al., 2001, Hämmerer et al., 2013). The impact of these confounding factors can be minimized by refraining from interpreting main effects of age such as absolute differences in neural activation, and instead focusing on, for example, age differences in within-person effects (cf. Rugg, 2017; Rugg and Morcom, 2005). Therefore, in the current analysis pipeline, we suggest comparing differences in the within-person item specificity effects (i.e., higher within-item similarity than between-item similarity) between age groups rather than absolute similarity values. Specifically, the clusters identified on the group level (second-level analysis) can be used to extract subject-specific similarity values (or effect sizes) at those channel × time × time coordinates that showed reliable differences, which can then be contrasted between groups. This approach is implemented in the current tutorial to investigate whether children and adults show differences in neural item specificity.

### 2.12 Implementation of third-level (between-group) analysis

**Fig. 7:**
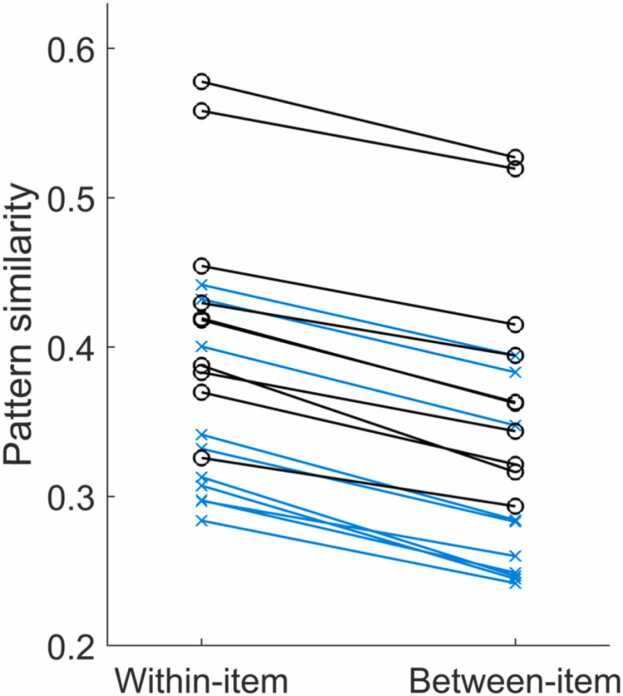
Comparison of mean within-item and between-item (within-category) pattern similarities extracted from identified clusters in individual children (blue, x) and adults (black, o). This figure can be created with step6_plot_sim_comparison.

Children’s and adults’ neural item specificity is contrasted in step4c_sim_comparison_3rd_level. For each group, all clusters from the 2nd level analysis with *p*s < 0.025 and the channel × time × time volume they enclose are identified and used as a mask to extract the individual within-item and within-category similarity values. These extracted similarities are averaged (see Fig. 7) and saved. Because the two age groups are compared with each other, the similarity data and statistics of both are required for this step.

**Fig. 8:**
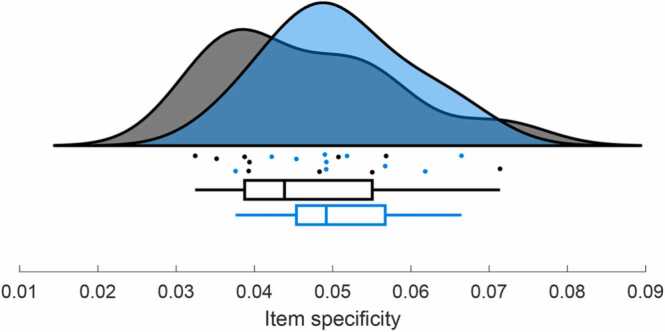
Item specificity (computed as the difference between within-item and within-category similarity) in children (blue) and adults (black). Group distributions as un-mirrored violin plots (probability density functions), boxplots with 1st, 2nd (median), and 3rd quartiles, whiskers with 2nd and 98th percentiles, and individual (vertically jittered) data points. This figure can be created with step6_plot_sim_comparison, which uses the raincloud_plot function (Allen et al., 2019).

The difference between within-item and within-category similarity (indicating item specificity) is computed for each subject, which is then used for the age group comparison that uses a standard two-sided independent samples *t*-tests to test for age difference in item specificity (see Fig. 8). The results are returned in the command window. In the provided sample dataset, children and adults do not show significantly different item specificity (*t* = 0.93, *p* = 0.364).

Instead of using a simple difference score, likewise, the individual effect sizes obtained in the first-level analysis can be extracted within the cluster masks and averaged (not implemented here). These would also indicate how much within-item and within-category similarity differ and thus how item-specific the neural representations are, and can then be compared between age groups.

### 2.13 Association with behavior

Our measure of item specificity reflects how much stimulus-specific information is represented in the neural activation patterns during stimulus encoding. According to previous research, adult participants, who show more item-specific neural representations, can better remember the items than participants with less item-specific representations can (Kobelt et al., 2021). The participants in the current dataset also performed a memory recognition test following the encoding phase. In the recognition task, exact item repetitions, similar lures (new exemplars from the same object categories), and entirely new objects were presented (cf. Stark et al., 2019), allowing for an estimation of precise item memory (for details, see Sommer et al., 2021). Here, as an example, we correlate item memory performance with neural item specificity (computed above) to identify between-person associations between brain and behavior. Other analyses to investigate behavioral effects of differences in neural specificity are possible, e.g., one could compute subsequent memory effects (Paller and Wagner, 2002), that refer to the within-person association of item specificity and the respective memory outcomes (see 3.8 Further applications).

### 2.14 Implementation

**Fig. 9:**
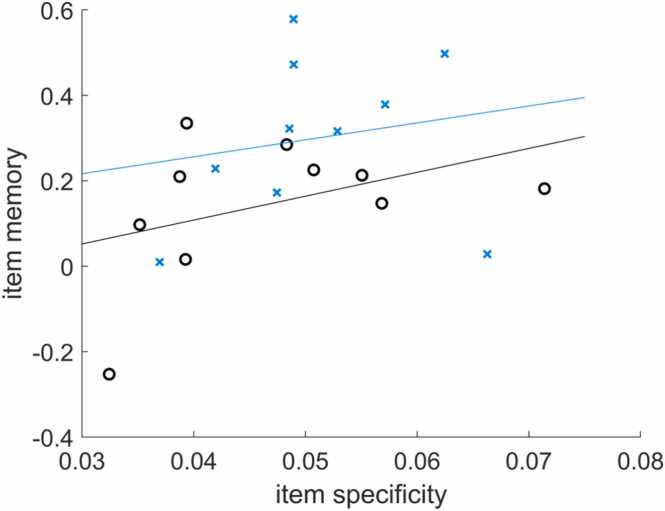
Between-subject association of item specificity and item memory in children (blue, x) and adults (black, o) indicated by least-squares lines. This figure can be created with step7_correlation_with_behavior.

Participants’ item memory performance is provided in the sample dataset (CH+YA_mean_item_memory.mat). The wrapper script step7_correlation_with_behavior loads the individual item specificity and item memory data and correlates them using Pearson correlations, separately for children and adults as well as across age groups. The results are returned in the command window. Furthermore, a scatter plot is created that illustrates the correlations (Fig. 9). Results from the sample dataset suggest that item memory and item specificity may be positively related to each other, but the correlations are not significant (children: *r* = 0.18, *p* = 0.612; adults: *r* = 0.41, *p* = 0.245; across groups: *r* = 0.35, *p* = 0.128).

### 2.15 Further applications

The current pipeline is just one example for the ways in which RSA can be used to compute the similarities of neural representations of certain stimuli, identify differences in these similarities, compare these differences between age groups, and associate them with behavior. We already hint on many more options above, and briefly list these and other applications that are feasible:

– **Category specificity** indicates how much category information is represented in the neural activation patterns and is measured as the difference between within-category and between-category similarity (Carp et al., 2011, Kobelt et al., 2021, Koen et al., 2019). It is one of the classic measures of aging-related neural dedifferentiation in fMRI, indicating that neural representations become less distinct in old age, but the evidence on its association to cognition is mixed (cf. Kobelt et al., 2021; Koen et al., 2020). The current pipeline can simply be adjusted for category specificity by replacing within-item and within-category similarity data by within-category and between-category similarity data, respectively (in all scripts for running the statistical comparisons as well as for plotting the results).

– **Pattern reinstatement** (or reactivation/replay; cf. Genzel et al., 2020) is the similarity of activation patterns during encoding to the activation patterns during retrieval, for example, in a recognition or recall task (Staresina et al., 2016, Wimber et al., 2012, Xue et al., 2010) and is thus closely related to pattern stability. It is a key element of episodic memory models that processes involved in an event’s encoding are also involved in its retrieval (Damasio, 1989, Norman and O’Reilly, 2003, Nyberg et al., 2000, Rugg et al., 2008). Reinstatement of item information has been demonstrated in temporal and spectral (intracranial) EEG patterns (Kerrén et al., 2018, Michelmann et al., 2018, Yaffe et al., 2014, Zhang et al., 2015). The current pipeline can be adjusted for pattern reinstatement analysis by using the self_rsa script with different data, namely encoding and retrieval data rather than first and second presentations during encoding.
– **Global similarity/matching** is the representational similarity among all stimuli that were presented in an experiment or condition (Davis et al., 2014, LaRocque et al., 2013, Lu et al., 2015, Sommer et al., 2019). Just like the implementation of between-category similarity, all pairwise comparisons are computed. Cognitive theories postulate that memory strength for an item arises from the similarity of its representation to the representations of other encoded items (Clark and Gronlund, 1996, Hintzman, 1984, Raaijmakers and Shiffrin, 1992, Xue, 2018). The current pipeline can be used to examine the association of global similarity and memory performance by computing between-item similarities separately for remembered and not remembered items and contrasting them with the scripts used for the statistical comparisons (see subsequent memory effects below). For an investigation of this effect in EEG time–frequency representations in young and older adults, see Sommer et al. (2019).
– **Representational dissimilarity matrix (RDM)** is an illustration of all pairwise item dissimilarities (inverse of similarities, e.g., correlation distance *r* – 1, or decodability) and thus characterizes the represented informational structure (Kriegeskorte et al., 2008). The rows and columns correspond to individual items and each cell is the (dis-)similarity between the two items (e.g., the averaged time–time similarity matrix). These RDMs can be compared to RDMs from other brain regions or other modalities, to hypothesized model RDMs, to behavior, and between individuals or species (i.e., second-order isomorphism; Edelman, 1998; Kriegeskorte et al., 2008). That is, in this step the represented informational structure is compared rather than the activity patterns themselves. The current pipeline can be used to run all pairwise item similarities (between-category similarity). The resulting time–time similarity matrices would need to be averaged to obtain one similarity value for each item pair in each participant, which could then be illustrated in an RDM and compared to other RDMs.
– **Subsequent memory effects** show within-person differences in neural activity in response to items that are subsequently remembered versus those that are not (Paller and Wagner, 2002). This can be applied to any measure of representational similarity by separating items according to their memory outcome for each participant before computing the individual similarity matrices. The resulting similarity matrices for remembered and not remembered items can then be contrasted using cluster-based permutation analysis (see Sommer et al., 2019).

### 2.16 Adjusting the code for your own data

The tutorial pipeline is specifically designed to analyze the provided dataset and needs adjustment to be applicable for other data formats. Since the pipeline depends on the FieldTrip toolbox and on its data structures, we would recommend that our readers also use FieldTrip’s preprocessing and/or frequency-decomposition tools, as these yield TFRs in the required format or that they convert their data to the FieldTrip format (e.g., for converting from EEGLAB, see: https://eeglab.org/others/EEGLAB_and_Fieldtrip.html). In addition to that, the trial selection in the current pipeline is specific to the sample dataset and the memory task paradigm. For application to your own data, you will also need to provide the specific trial information, e.g., about item repetitions and category membership. This information is used in self_rsa and betw_cat_rsa and needs to be adjusted (in the code section "Find stimulus pairs") to select those trials that are to be correlated.

## 3 Challenges and limitation

In this tutorial we argue that the investigation of neural representations may open new avenues to understand developmental changes in cognition across childhood, and suggest that pattern similarity analysis of time-resolved brain recordings (such as EEG) provides a powerful tool to delineate developmental differences in the temporal dynamics of neural representations (e.g., Fellner et al., 2020; Jafarpour et al., 2013; Sommer et al., 2019). The differences between classic univariate and multivariate analyses are often interpreted as focusing on different neurocognitive aspects, namely on the involvement of a particular region in a function versus the representational content present in that region (Mur et al., 2009). However, we would like to advise caution in claiming that univariate versus multivariate analyses measure neural processes versus neural representations as these may not be strictly separable (cf. Davis and Poldrack, 2013; Xue, 2018). Indeed, the neural activation patterns that we define as neural representations of course also capture neural processes. Furthermore, the dichotomy between process and content is, like the computer metaphor of the brain in general, useful for cognitive theories but does not actually reflect the biology of neural systems (Feldman, 2016, Searle, 1990). As the underlying neural units are recurrently connected in local and global networks (e.g., Bullmore and Sporns, 2009; van den Heuvel and Hulshoff Pol, 2010), any activity of a given neural assembly that represents a certain mnemonic content will also immediately lead to its transformation within the context of ongoing network activity. This transformation can be regarded as a process, emphasizing the practical inseparability of representations and processes as understood in the cognitive literature (e.g., Tulving and Bower, 1974). Hence, although one can focus on the representational aspects of the neural basis of cognition rather than on specific neural processes, neural representations are not strictly to be understood as being separate from processes such as encoding (cf. Feldman, 2016). Currently, in cognitive neuroscience, representations are a central concept that link cognition to brain activity (Kriegeskorte and Kievit, 2013), and do not necessarily distinguish between content and process. Neural activity represents content and, at the same time, reflects the processes concerned with these contents. Nonetheless, multivariate similarity analyses are able to differentiate content-specific information (e.g., Kuhl and Chun, 2014), even at the level of individual stimuli (Kobelt et al., 2021, Yaffe et al., 2014, Zheng et al., 2018) and are thus suitable to study neural representations in a broader sense.

In the methods introduced in this tutorial, we focus on dynamic neural representations and their fine-grained similarities across trial times. Traditionally, neural representations are interpreted and studied as spatially distributed activation patterns and accordingly usually measured with fMRI (e.g., Haxby et al., 2001; Rissman and Wagner, 2012). While the spatial resolution of EEG is much lower than of fMRI, and spatial differences of neural representations are therefore not the main focus of EEG-based RSA, the high spatial resolution in fMRI comes at the cost of low temporal resolution. Hence, fMRI does not lend itself to techniques like the presented time–time similarity computations. Here, we exploit the rich information contained across multiple neural rhythms and topographical sites by applying RSA to time–frequency-transformed EEG data, which allows identification of item-specific neural signatures (Kerrén et al., 2018, Michelmann et al., 2016, Staresina et al., 2016, Zhang et al., 2015, Sommer et al., 2019). Our approach may be particularly attractive for developmental researchers, as among all available neuroimaging tools, EEG is probably most readily available and highly feasible to collect high-quality data from children and even infants, at the lowest cost. Overall, different brain recording and pattern similarity approaches offer complementary views on neural representational properties and their individual strengths should be combined by integrating results from different methodologies to gain a complete picture of how the brain encodes information.

## 4 Conclusions

Time-resolved spectral pattern similarity analysis provides a powerful toolset for cognitive neuroscientists to gain insights into what and how information is dynamically represented in the human brain. In this article, we provide a thorough introduction and tutorial for applying multivariate similarity analysis on EEG data, based on time–frequency power spectra. To provide a practical example, we implemented an executable pipeline from single-subject similarity analyses at different representational levels to statistical comparisons using non-parametric cluster-based permutation tests, explaining each step and discussing practical considerations and alternative approaches. We expect multivariate similarity analyses to have an increasing impact on developmental cognitive neuroscience, opening up new research directions and enhancing our understanding of how the contents and properties of neural representations develop over the lifespan and influence cognitive abilities. Our tutorial makes advanced multivariate EEG pattern similarity analyses readily accessible to a broad audience of developmental scientists, facilitating the wider adoption of such approaches.

## Ethics approval statement

All experiments were approved by the respective local ethics committee.

## Declaration of Competing Interest

The authors declare that they have no known competing financial interests or personal relationships that could have appeared to influence the work reported in this paper.

## Data Availability

Code and data are publicly available: https://osf.io/jbrsa/.
